# Compare the physicochemical and biological properties of engineered polymer-functionalized silver nanoparticles against *Porphyromonas gingivalis*

**DOI:** 10.3389/fmicb.2022.985708

**Published:** 2022-09-08

**Authors:** Meng Zhang, Edward C. M. Lo

**Affiliations:** Faculty of Dentistry, The University of Hong Kong, Hong Kong, Hong Kong SAR, China

**Keywords:** antibacterial, antibiofilm, cytotoxicity, polymers, *Porphyromonas gingivalis*, silver nanoparticles

## Abstract

**Background:**

Some polymer-functionalized AgNPs (P-AgNPs) have been developed to optimize the biological properties of AgNPs. However, there are no studies in the literature comparing the differences in physicochemical and biological properties of AgNPs caused by various polymer-functionalizations and providing evidence for the selection of polymers to optimize AgNPs.

**Methods:**

Two AgNPs with similar nano-size and opposite surface charges were synthesized and functionalized by seven polymers. Their physicochemical properties were evaluated by UV-Visible absorption, dynamic light scattering, transmission electron microscopy and inductively coupled plasma optical emission spectroscopy. Their biological properties against *Porphyromonas gingivalis* and human gingival fibroblast were investigated by MIC determination, time-dependent antibacterial assay, antibiofilm activity and cell viability assay. Silver diamine fluoride, AgNO_3_ and metronidazole were used as positive controls.

**Results:**

Comparative analysis found that there were no significant differences between P-AgNPs and AgNPs in nano-size and in surface charge. Raman spectroscopy analysis provided evidence about the attachment of polymers on AgNPs. For antibacterial property, among the negatively charged AgNPs, only polyvinylpyrrolidone (PVP)-functionalized AgNPs-1 showed a significant lower MIC value than AgNPs-1 (0.79 vs. 4.72 μg/ml). Among the positively charged AgNPs, the MIC values of all P-AgNPs (0.34–4.37 μg/ml) were lower than that of AgNPs-2 (13.89 μg/ml), especially PVP- and Pluronic127-AgNPs-2 (1.75 and 0.34 μg/ml). For antibiofilm property, PVP-AgNPs-1 (7.86 μg/ml, *P* = 0.002) and all P-AgNPs-2 (3.42–31.14 μg/ml, *P* < 0.001) showed great antibiofilm effect against *P. gingivalis* biofilm at 5* to 10*MIC level. For cytotoxicity, all negatively charged AgNPs and PVP-AgNPs-2 showed no cytotoxicity at MIC level, but significant cytotoxicity was detected at 2.5* to 10*MIC levels.

**Conclusion:**

Among the polymers studied, polymer functionalization does not significantly alter the physical properties of AgNPs, but modifies their surface chemical property. These modifications, especially the functionalization of PVP, contribute to optimize the antibacterial and antibiofilm properties of AgNPs, while not causing cytotoxicity at the MIC level.

## Introduction

Silver ion is an antibacterial metal ion with a long history of application (Barras et al., 2018). With the advance of nanoscience and nanotechnology, silver nanoparticles (AgNPs) have been developed to provide a wider variety of physical, chemical and biological properties (Bhattacharya and Mukherjee, 2008). The antibacterial efficacy and bioavailability of AgNPs has been confirmed by research (Chernousova and Epple, 2013; Konop et al., 2016). AgNPs are powerful nano-weapons against bacteria, including multidrug-resistant bacteria, and possess broad application potential (Rai et al., 2012). Some AgNPs products have been applied in biomedical field (Chen and Schluesener, 2008).

However, the safety issue caused by the exposure and accumulation of silver in the environment and in the human body cannot be ignored and has received increasing attention. Due to the large surface area and intrinsic physicochemical properties of nanomaterials, some studies reveal that AgNPs have different interaction modes with living cells and different antibacterial mechanisms compared with silver ions, (Kȩdziora et al., 2018; Malysheva et al., 2021). However, the toxicity mechanism of AgNPs in cytoplasm is still primarily correlated with the release of silver ions (Lok et al., 2006). Hence, in order to reduce the accumulation and toxicity of silver, many studies have been devoted to decrease the working concentration of silver by optimizing AgNPs’ biological properties (Makvandi et al., 2021).

The biological properties of AgNPs are closely associated with their surface physicochemical properties, including nano dimension, shape, surface charge, colloidal state, and surface chemical (Xiu et al., 2012; Dakal et al., 2016). Polymer functionalization is an effective way to modify the surface physicochemical properties of AgNPs and optimize their biological properties (Hajtuch et al., 2019; Huma et al., 2020). Polyethylene glycol (PEG), Polyvinylpyrrolidone (PVP) and Pluronic™ are representative and frequently used polymers in research (Bryaskova et al., 2011; Rolim et al., 2019; Tepale et al., 2019). Both PEG and PVP are considered biologically safe, and have been applied in various fields, including biomedical, industry and personal care. Their functionalization for enhancing the antibacterial and antibiofilm activities of AgNPs has been explored (Tiwari et al., 2017; Zhao et al., 2017). Some studies detected a lower cytotoxicity instead of a better antibacterial activity for polymer-functionalized AgNPs (Selvakumar et al., 2018; Barrios-Gumiel et al., 2019). In addition, Pluronic™, a group of non-ionic poly (ethylene oxide, PEO)-poly (propylene oxide, PPO)-PEO triblock copolymers with various molecular weights, has excellent surfactant properties in dispersion, stabilization and emulsification aspects and good biocompatibility (Alexandridis and Alan, 1995). These polymers may be used to modify AgNPs so as to boost stability, as well as antibacterial and antibiofilm effectiveness (dos Santos et al., 2012; Liu et al., 2021). However, it is not known which polymers can better optimize the biological properties of AgNPs. So far, there are no studies comparing the effectiveness of different polymer-functionalization on improving the physicochemical and biological properties of AgNPs. Therefore, in the present study seven commonly used polymers which have different hydrophilic-hydrophobic property, excellent surface activity and different molecular weights were selected to explore the above question.

**Scheme 1:**
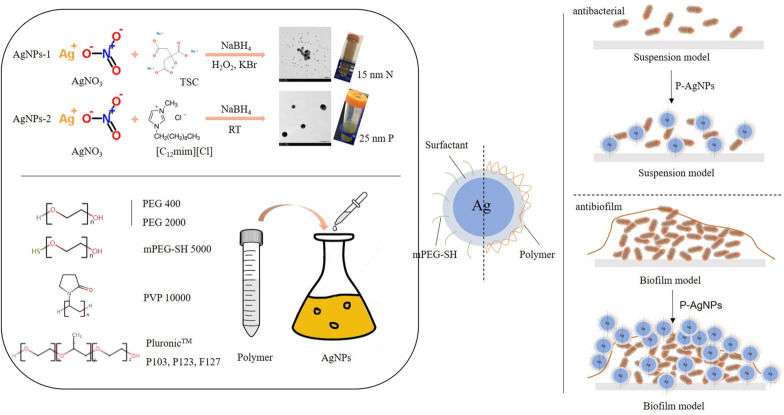
Two silver nanoparticles (AgNPs) with similar dimension and opposite surface charges were synthesized and functionalized with seven polymers, and their physical and biological properties (including antibacterial activity, antibiofilm activity, and cytotoxicity) were subsequently detected.

In dentistry, *Porphyromonas gingivalis* (*P. gingivalis*) is a crucial pathogen of periodontitis, and it is also associated with some systemic diseases, such as type 2 diabetes, cardiovascular disease, Alzheimer disease and aspiration pneumonia (D’Aiuto et al., 2018; Park et al., 2019; Bulgart et al., 2020; Khadka et al., 2021). Inhibition of *P. gingivalis* is important for reducing the risk of periodontitis and the above-mentioned systemic diseases. Therefore, inhibition of *P. gingivalis* was used in the present study for comparing the biological properties of the different polymer-functionalized AgNPs. In this study, we synthesized two types of AgNPs with similar nano dimensions but opposite surface charges, and then functionalized them using seven polymers with different molecular weights (PEG, PVP, and Pluronic™). This study aimed to compare the physical and biological properties of the engineered polymer-functionalization AgNPs, and to identify a polymer that can greatly optimize the biological property of AgNPs against *P. gingivalis* (Scheme 1).

## Materials and methods

### Synthesis of nano silver

Silver nanoparticles with similar size but opposite surface charges were synthesized based on previously reported methods.

Negatively charged AgNPs – based on Vladimir’s method with slight modification (Frank et al., 2010), the following freshly prepared solutions were mix together in the following order: 7 ml of 12.5 mM Trisodium citrate (TSC), 17.5 ml of 0.375 mM silver nitrate (AgNO_3_), 17.5 ml of 10 mM hydrogen peroxide (H_2_O_2_), and 228 μl of 10 mM potassium bromide (KBr). Then 8 ml of 5 mM sodium borohydride (NaBH_4_) was added dropwise into the above solution until there was no further visible color change, and the product was stirred for 2 h at room temperature. The brown-red product solution was the study negatively charged AgNPs (named AgNPs-1 in this study).

Positively charged AgNPs – based on Abbaszadegan’s method with slight modification (Abbaszadegan et al., 2015a), 1 ml of 10 mM AgNO_3_ was dropped into 20 ml of 6.2 mM 1-dodecyl-3-methylimidazolium chloride ([C12mim][Cl]) aqueous solution and vigorously stirred. Then 100 μl of freshly prepared 0.4 M NaBH_4_ was dropped into the resultant solution to obtain a golden solution of positively charged AgNPs (named AgNPs-2 in this study).

### Synthesis of polymer-functionalized AgNPs (P-AgNPs)

PEG, PVP, and Pluronic™ with different molecular weights, including PEG 400, PEG 2000, PEG-SH 5000, PVP (MW 10000), Pluronic P103 (Mn 5000), P123 (Mn 5800), and F127 (Mn 12000), were used to functionalized the synthesized AgNPs. The molar ratio of Ag ion and polymer PEG/PVP was set from 1:1 to 1:10 (see Supplementary Table 1; Bryaskova et al., 2011; Rolim et al., 2019; Tepale et al., 2019), and the polymers were freshly pre-dissolved in 10 ml of sterile DI (deionized) water. The Pluronic™ products were directly added to the AgNPs solutions at the value lower than the CMC (critical micelle concentration) value, while an additional 10 ml of sterile DI water was added for homogenization. The CMC value of P103, P123, and F127 were 0.1, 0.052 and 1 mg/ml, respectively. Polymer solution/powder of appropriate concentration and weight was added to the corresponding synthesized AgNPs solution to encapsulate the AgNPs (the detailed doses used are shown in Supplementary Table 1), and then the product solutions were stirred overnight for full reaction.

All AgNPs solutions were centrifuged and washed three times at 15,000 rpm for 15 min each to remove the excess unreacted ionic liquid. The pellet was then resuspended in 200 μl of sterile DI water, and the AgNPs-1/2 and P-AgNPs-1/2 products were stored at room temperature.

### The physicochemical properties of AgNPs

UV-Visible (UV-Vis) absorption. The 100 × diluted solutions of AgNPs in sterile DI water were prepared to detect the UV-Vis absorption spectroscopy (200–700 nm, 10 nm interval).

The silver concentration of AgNPs was determined by ICP-OES (inductively coupled plasma optical emission spectroscopy). Briefly, 1 μl of AgNPs was added to 9 μl of 68% nitric acid to convert the silver element into silver ion. Subsequently, 3.99 ml of 1 M nitric acid solution was used to dilute the silver ion solution. Thus, 4000-fold diluted solutions of AgNPs were analyzed using ICP-OES.

A series of 10× to 100× diluted solutions of AgNPs in sterile DI water (*ca*. 20 μg/ml) were prepared for the determination of hydrated size and zeta potential of AgNPs using dynamic light scattering (DLS) (Malvern, Nano-zetasizer).

The morphology and size of AgNPs were detected by transmission electron microscopy (TEM, Philips CM100). Ten microliters of 10× to 100× diluted solutions of AgNPs in sterile DI water were deposited onto a 400-mid formvor/carbon-coated copper grid. The samples were put on filter paper and dried at room temperature for 1 h, and then images of the AgNPs were captured using TEM.

Raman spectroscopy was used to detect qualitatively the attachment of polymers on the surface of AgNPs (Cristóbal-García et al., 2018). Briefly, 5 μl of each sample was pipetted on a 5 × 5 mm silicon disc which performed as substrate. All samples were placed in a biological safety cabinet and dried for 2 h. Raman spectra were collected with WITEC (alpha300R) confocal Raman system. The spectra were scanned between 100 and 3,500 cm^–1^ with wavelength 532 nm, and 100× objective magnification was used for data acquisition. The exciting power was *ca.* 10 mW and at least ten repetitions were performed. All Raman spectroscopy measurements were conducted at 25°C.

### Antibacterial effect

The minimum inhibitory concentration (MIC) for AgNPs against planktonic *P. gingivalis* was determined using the microdilution method based on the Clinical Laboratory Standards Institute (CLSI) guideline (Pfaller and Diekema, 2012). The maximum test concentration of AgNPs for MIC determination was set to be 2% of the volume of the bacterial suspension. In brief, 100 μl of *P. gingivalis* broth containing serially diluted AgNPs were pipetted into a 96-well cell plate. Subsequently, 10^8^ CFU/ml (OD_660*nm*_ = 0.271–0.279) of *P. gingivalis* in the late logarithmic phase was prepared and inoculated into fresh *P. gingivalis* broth (ratio = 2:100). Then 100 μl of the prepared *P. gingivalis* bacterial suspension (10^6^ CFU/ml) was pipetted into the 96-well cell plate and inoculated in an anaerobic incubator at 37°C for 3 days. Absorbance values at OD_660*nm*_ were recorded to assess the growth of *P. gingivalis*. Negative control and positive controls, including 38% silver diamine fluoride (SDF, Japan), AgNO_3_, and metronidazole (MNZ), were set up parallelly, and three parallel samples and three independent replicates were set up to determine the MIC of AgNPs. The MIC was defined as the lowest concentration that substantially inhibited bacteria growth in the medium.

### Time-dependent antibacterial effect of AgNPs

In a 96-well cell plate, 200 μl/well of *P. gingivalis* bacterial suspensions (10^6^ CFU/ml) were prepared and then shocked by AgNPs at MIC level in an anaerobic incubator at 37°C for 30 min, 2, 6, and 24 h. Then 5 microliters of suspension were dropped on blood agar and incubated for 5 days. Three independent assays were performed in parallel. Photographs of the blood agar were taken with a camera.

### Antibiofilm effect of AgNPs on *Porphyromonas gingivalis* biofilm

The mature *P. gingivalis* biofilms were incubated in iBidi Cell Plate which was specifically applied for confocal laser scanning microscopy (CLSM). Briefly, 200 μl of *P. gingivalis* suspension (10^8^ CFU/ml) in fresh *P. gingivalis* broth was placed in iBidi Cell Plate and incubated in an anaerobic incubator at 37°C for 6 days (*P. gingivalis* broth was refreshed every 3 days). When the mature *P. gingivalis* biofilms were constructed, we gently discarded the supernatant in the iBidi cell plate and pipetted 200 μl of fresh *P. gingivalis* broth containing AgNPs at MIC and 10*MIC concentration into the cells. After an extra 24 h incubation in anaerobic incubator, we removed the supernatant, washed the cells with PBS and stained the biofilm with SYTO9 and PI dyes (Live/Dead bacterial viability kit, Thermo Fisher) for 30 min. The fluorescence images were captured by the CLSM.

In addition, the dose-dependent bactericidal effect of AgNPs and P-AgNPs on mature *P. gingivalis* biofilm was detected using CCK8 assay. The mature *P. gingivalis* biofilms were prepared and incubated in a 96-well plate. In short, 100 μl of 10% FBS per well was pre-cultured in a sterile 96-well plate for 1 h, 80 rpm at 37°C. After removing the FBS, we added 100 μl of fresh *P. gingivalis* broth containing 1% of *P. gingivalis* suspension (10^8^ CFU/ml) to each well, and incubated the plate in an anaerobic incubator for 6 days at 37°C, with the medium changed every 3 days. Subsequently, we replaced the suspension with fresh medium and performed turbidity measurements at OD600 nm (initial value) to confirm the consistency of mature biofilm. Sterile media containing AgNPs-1/2 and P-AgNPs-1/2 with MIC, 2.5*MIC, 5*MIC, 7.5*MIC and 10*MIC concentrations were freshly prepared at the same time, and were pipetted into the well plate. After incubation for another 24 h, the suspension was replaced by fresh medium containing 10% CCK8 and the optical density (at 450 nm) of the wells was detected in 2 h.

### Cytotoxicity of AgNPs

Human gingival fibroblasts (HGF-1, P6) were used to detect the cytotoxicity of the AgNPs. HGF-1 (P3) were resuscitated and passaged in Dulbecco’s modified eagle medium (DMEM) containing 10% fetal bovine serum (FBS), 100 U/ml penicillin, and 100 μg/ml streptomycin. Subsequently, 5,000 cells/well (P6) were seeded in a 96-well cell plate. After incubation for an additional 24 h for cell attachment, cells were shocked with fresh medium containing AgNPs at MIC for 6, 24, and 48 h. The medium was then removed and replaced with 90 μl of PrestoBlue Cell viability reagent (10% in PBS, Thermo Fisher.) for 10 min at 37°C. The fluorescence in 560/590 nm (excitation/emission) were read by SpectraMax M2 (United States). Five parallel samples and three independent assays were performed.

### Statistical analysis

Unless otherwise specified, all the graphs were drawn with GraphPad Prism 5 software. The data were analyzed using the statistics software SPSS V21.0 (IBM Corporation, Armonk, NY, United States). Normality of data distribution was assessed by Shapiro–Wilk test. Differences between groups were assessed by *t*-test and one-way ANOVA with Bonferroni multiple comparison tests. A *p*-value at or less than 0.05 was regarded as statistically significant.

## Results

### The physicochemical properties of AgNPs-1/2 and P-AgNPs-1/2

**FIGURE 1:**
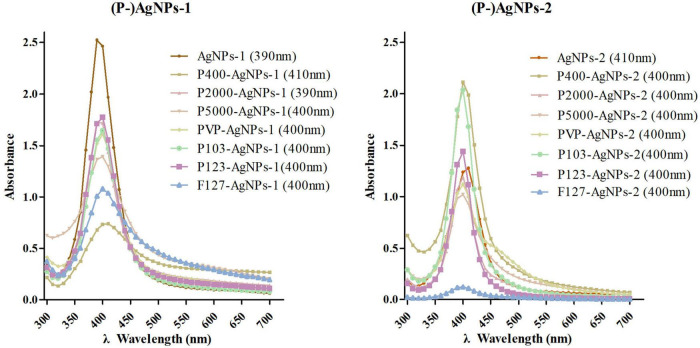
The UV-Vis absorption spectroscopy detection of AgNPs-1/2 and P-AgNPs-1/2. In parentheses is the peak value of the samples.

**FIGURE 2:**
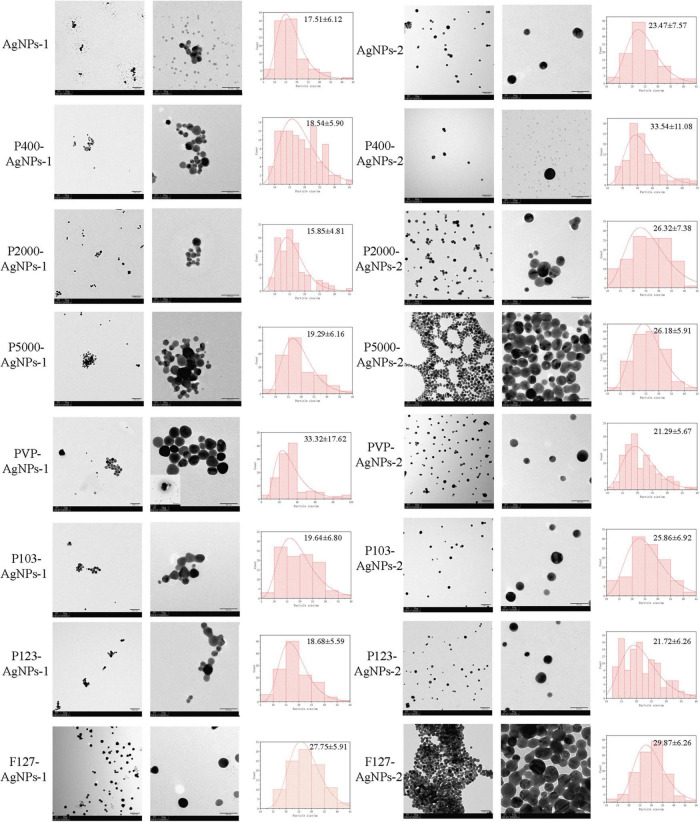
TEM images of AgNPs-1/2 and P-AgNPs-1/2, along with the histograms of nanometer size distribution (ImageJ). The scale bar values for each pair of images are 100 nm **(left)** and 50 nm **(right)**.

AgNPs-1 and AgNPs-2 were successfully synthesized. The UV-Vis absorption spectra detection showed that the peak values of these two AgNPs were 390 and 410 nm, respectively (Figure 1). The hydrated size was confirmed by DLS (Table 1). The mean and SD values of AgNPs-1 and AgNPs-2 were 15.70 ± 2.26 nm and 24.40 ± 4.16 nm, respectively. TEM image results (Figure 2) showed a spherical morphology of AgNPs-1/2 and the respective sizes were 17.51 ± 6.12 nm and 23.47 ± 7.57 nm, which were consistent with the results of DLS detection. In addition, the zeta-potential (ζ-potential) of AgNPs-1 as detected by DLS was −36.3 mV, that of AgNPs-2 was + 46 mV (Table 1). The silver concentration of the two AgNPs products, were 0.944 and 3.473 mg/ml (Table 1).

**TABLE 1:** Summary of the nano size, zeta-potential (DLS), silver concentration (ICP-OES), and MIC values for AgNPs-1/2 and P-AgNPs-1/2.

Code			Size			Zeta-potential		AgNPs concentration (mg/ml)	MIC (μg/ml)
			Mean (d.nm)	SD	PDI	Mean (mV)	SD		
SDF									9.00
AgNO3									0.90
Metronidazole									0.13
AgNPs-1			15.7	2.256	0.149	–35.0	3.170	0.944	4.72
	a-	PEG400	50.8	2.783	0.242	–10.7	0.700	0.302	6.03
	b-	PEG2000	105.7	1.708	0.471	–30.5	1.150	1.225	6.13
	c-	PEG-SH 5000	105.7	3.190	0.384	–18.0	0.702	1.077	3.37
	d-	PVP	122.4	2.219	0.256	–19.6	0.833	1.179	0.79
	e-	P103	78.8	0.760	0.419	–31.9	5.450	0.728	4.86
	f-	P123	73.0	0.331	0.393	–31.5	6.650	0.711	3.56
	g-	F127	105.7	10.340	0.273	–28.6	0.265	0.623	4.15
AgNPs-2			24.4	4.158	0.300	46.0	4.440	3.473	13.89
	a-	PEG400	117.4	3.365	0.379	25.7	0.929	4.370	4.37
	b-	PEG2000	68.1	2.986	0.264	38.1	1.460	0.991	1.98
	c-	PEG-SH 5000	122.4	5.164	0.310	20.2	2.690	2.560	2.56
	d-	PVP	105.7	2.420	0.345	27.4	0.967	1.750	1.75
	e-	P103	58.8	1.050	0.240	22.5	3.700	2.076	4.15
	f-	P123	50.8	9.646	0.198	44.0	0.971	1.418	2.84
	g-	F127	58.8	1.018	0.269	28.7	2.790	0.171	0.34

SDF, silver diamine fluoride; SD, standard deviation; PDI, polydispersity index; MIC, minimum inhibitory concentration.

The AgNPs-1/2 functionalized by PEG400, PEG2000, PEG-SH5000, PVP, Pluronic P103, P123, F127 were characterized by the above tests as well. The UV-Vis absorption spectrum peaks of all polymer-functionalized AgNPs-1/2 (P-AgNPs-1/2) were between 390 and 410 nm (Figure 1). The DLS results (Table 1) show that the hydrated particle sizes of P-AgNPs-1/2 were between 50 and 130 nm, which were all larger than the sizes of AgNPs-1/2. However, the TEM image showed that the particle size of all P-AgNPs remained below 40 nm and did not increase significantly (Figure 2).

All the P-AgNPs-1 were negatively charged, with ζ-potential between −10.7 and −31.9 mV. All the P-AgNPs-2 were positively charged, with ζ-potential between 20.2 and 44.0 mV (Table 1). The silver concentrations of P-AgNPs-1/2 were 0.302–1.225 mg/ml and 0.171–4.37 mg/ml, respectively (Table 1).

**FIGURE 3:**
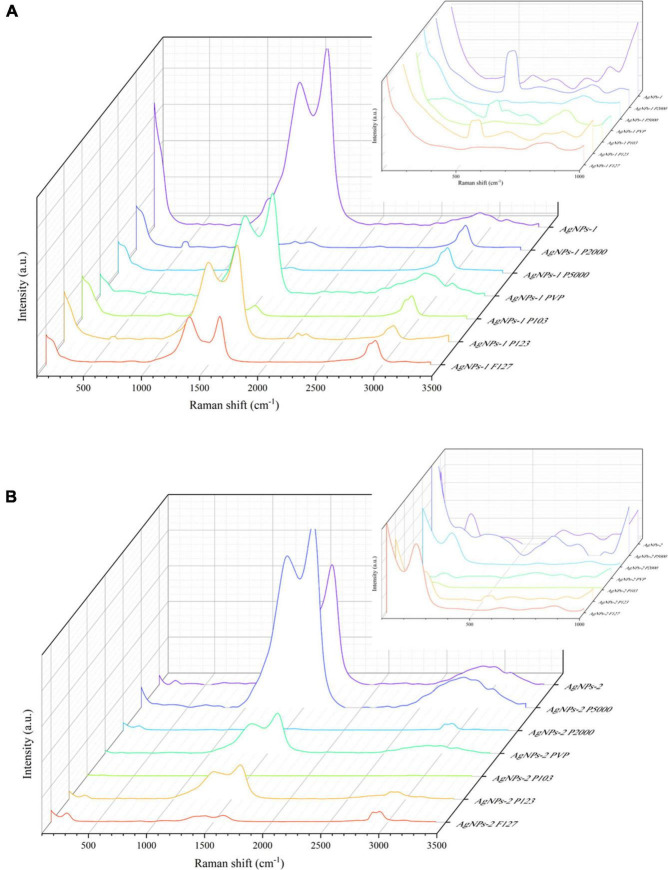
Raman spectrum of AgNPs-1/2 and P-AgNPs-1/2. **(A)** AgNPs-1 and P-AgNPs-1. **(B)** AgNPs-2 and P-AgNPs-2. The inset figures are corresponding zoomed-in views at 100–1,000 cm^– 1^.

Raman spectra of the polymers and the (P-)AgNPs are shown in Supplementary Figure 1 and Figure 3. The peaks at 517 and 964 cm^–1^ came from substrate silicon. AgNOs showed a typical band of nitrate ion at 1,045 cm^–1^, polymers showed some typical bands at *ca.* 1,200, 1,400 and 2,900 cm^–1^ (see Supplementary Figure 1A). Both AgNP-1 and AgNPs-2 showed two widened bands at 1,351, 1,584 cm^–1^ and 1,364, 1,584 cm^–1^, respectively. AgNPs-2 showed a sharp band at 234 cm^–1^ but AgNPs-1 did not (Figures 3A,B). After undergoing polymer-functionalization, PEG-SH 5000 functionalized AgNPs showed a typical band at ca. 400 cm^–1^. PVP functionalized AgNPs showed a typical band at ca. 650 cm^–1^. Pluronic P103 functionalized AgNPs-1/2 showed a typical band at ca. 2,200 and 3,200 cm^–1^, respectively. All P-AgNPs-1 showed strong and broad bands at ca. 500 and 2,900 cm^–1^ (Figures 3A,B and Supplementary Figure 1B).

### Antibacterial effect of AgNPs-1/2 and P-AgNPs-1/2 on *Porphyromonas gingivalis* suspension

The MIC values of AgNPs-1/2 and P-AgNPs-1/2 against *P. gingivalis* suspension are shown in Table 1. The MIC values of AgNPs-1/2 were 4.72 and 13.89 μg/ml, respectively. Among the AgNPs-1 and P-AgNPs-1 subgroups, only functionalization with PVP (MIC: 0.786 μg/ml vs. AgNPs-1: 4.72 μg/ml) greatly improved the antibacterial capability of AgNPs-2, and the antibacterial effects of other subgroups were similar to that of AgNPs-1 (MIC: 3.37 to 6.13 μg/ml vs. 4.72 μg/ml). Among the AgNPs-2 and P-AgNPs-2 subgroups, all P-AgNPs-2 subgroups significantly improved the antibacterial effect of AgNPs-2 (0.34 to 4.37 μg/ml vs. 13.89 μg/ml). Functionalization with P2000 (1.98 μg/ml), PVP (1.75 μg/ml) and F127 (0.34 μg/ml) greatly improved the antibacterial performance of AgNPs-2.

**FIGURE 4:**
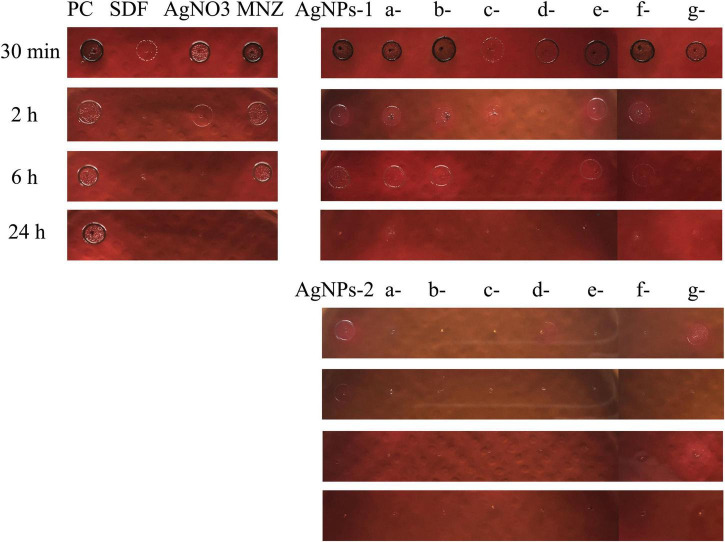
Summary for the time-dependent antibacterial effect of AgNPs-1/2 and P-AgNPs-1/2 on *P. gingivalis* suspensions. a, b, c, d, e, f, and g are PEG 400, PEG 2000, PEG-SH 5000, PVP (MW 10000), Pluronic P103 (Mn 5000), P123 (Mn 5800), and F127 (Mn 12000), respectively. SDF, silver diamine fluoride; MNZ, metronidazole.

The time-dependent antibacterial effect of AgNPs is shown in Figure 4. In AgNPs-1 and P-AgNPs-1 subgroups, P-SH5000- and PVP-AgNPs-1 showed an obvious inhibitory effect at 30 min, P-SH5000- showed complete bactericidal effect at 6 h, and PVP-AgNPs-2 showed complete bactericidal effect at 2 h, similar to the SDF control. F127-AgNPs-2 also showed complete bactericidal effect at 2 h but no effect at 30 min. Other subgroups showed significant inhibitory effect at 2 h and complete bactericidal effect at 24 h, which is similar to the MNZ group. In AgNPs-2 and P-AgNPs-2 subgroups, P400-, P2000-, P- SH5000-, PVP-, P103- and P123- AgNPs-2 completely inhibited the growth of *P. gingivalis* suspensions at 30 min, which was better than the SDF control group. AgNPs-2 and F127-AgNPs-2 showed significant inhibitory effect at 30 min and complete bactericidal effect at 2 h which was similar to that of SDF.

### Antibiofilm effect of AgNPs and p-AgNPs on mature *Porphyromonas gingivalis* biofilm

**FIGURE 5:**
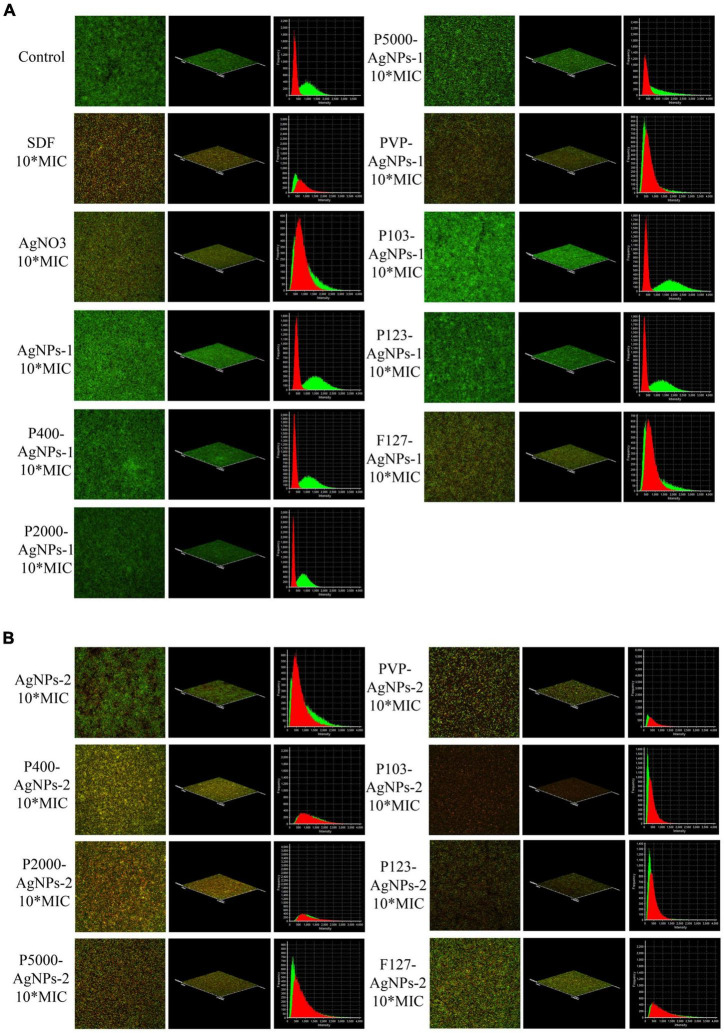
CLSM images at 60× magnification show the antibiofilm effect of **(A)** SDF, AgNO3, AgNPs-1, P-AgNPs-1 and **(B)** AgNPs-2 and P-AgNPs-2 on *P. gingivalis* biofilm (Live/Dead bacterial viability kit), including merged images, 3D models, and intensity-frequency area graphs. Red for dead bacteria and green for live bacteria.

The live/dead bacteria staining test results are summarized in Figure 5. The *P. gingivalis* biofilms were not invaded and destroyed by any of the AgNPs and P-AgNPs groups at MIC level (data not shown). Despite this, some P-AgNPs-1 and (P-)AgNPs-2 subgroups at 10*MIC level were able to kill *P. gingivalis* in biofilm, as reflected by the large amount of red fluorescence shown in the images in Figure 5. These were the PVP-AgNPs-1 (7.8 μg/ml), F127-AgNPs-1 (41.5 μg/ml), AgNPs-2 (138.9 μg/ml) and all P-AgNPs-2 subgroups (3.4 –43.7 μg/ml).

**FIGURE 6:**
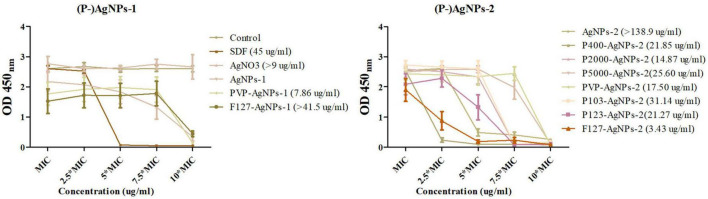
The dose-dependent antibiofilm effect of AgNPs-1/2 and P-AgNPs-1/2 on *P. gingivalis* biofilm (CCK8 kit). In parentheses is the minimize antibiofilm concentration of AgNPs-1/2 and P-AgNPs-1/2.

In addition, in order to determine the dose-dependent bactericidal effect of the above subgroups against *P. gingivalis* biofilm, cell viability (CCK8 assay) test of AgNPs-2 and P-AgNPs-1/2 subgroups at MIC, 2.5*MIC, 5*MIC, 7.5*MIC and 10*MIC level on *P. gingivalis* biofilm were performed (Figure 6). The initial value of constructed *P. gingivalis* biofilm at OD_600*nm*_ was 0.22 ± 0.03, and no significant difference was detected. A *P. gingivalis* biofilm with consistent growth condition was successfully constructed. In the results obtained after treatment with the various study AgNPs, SDF, AgNO_3_ (positive control) or DI water (negative control), no significant differences in OD450 values were detected between the (P-)AgNPs-1/2 subgroups and the negative control group at the MIC level, except for the PVP-AgNPs-1 (*P* = 0.018), F127-AgNPs-1 (*P* = 0.010) and F127-AgNPs-2 (*P* = 0.034) subgroups. As positive controls, SDF at 5*MIC level (45 μg/ml) displayed significant bactericidal effect (*P* < 0.001) while AgNO_3_ showed a significant bactericidal effect at 10*MIC level (9 μg/ml, *P* < 0.001). In the AgNPs-1 and P-AgNPs-1 subgroups, PVP-AgNPs-1 (7.86 μg/ml) and F127-AgNPs-1 (41.50 μg/ml) showed a significant bactericidal effect (*P* = 0.002 and 0.011) at 10*MIC level compared with the MIC level. In the AgNPs-2 and P-AgNPs-2 subgroups, AgNPs-2 at 5*MIC (69.45 μg/ml) to 10*MIC level (138.9 μg/ml) showed a significant bactericidal effect (*P* < 0.001). P400-AgNPs-2 had a significant bactericidal effect at 2.5*MIC level (10.93 μg/ml, *P* < 0.001), and more significant bactericidal effect (OD_450*nm*_ < 0.10) at 5*MIC level (21.85 μg/ml, *P* < 0.001). P2000-AgNPs-2 (14.87 μg/ml, *P* < 0.001), P103-AgNPs-2 (31.14 μg/ml, *P* < 0.001) and P123-AgNPs-2 (21.27 μg/ml, *P* < 0.001) showed significant bactericidal effects at 7.5*MIC. P-SH5000-AgNPs-2 (25.60 μg/ml, *P* < 0.001), PVP-AgNPs-2 (17.50 μg/ml, *P* < 0.001), and F127-AgNPs-2 (3.43 μg/ml, *P* = 0.001) showed significant bactericidal effects at 10*MIC. However, although the bactericidal effects in AgNO_3_, PVP-AgNPs-1, F127-AgNPs-1 and AgNPs-2 subgroups were statistically significant at 10*MIC level, their OD_450*nm*_ values were significantly higher than that of the control.

A 100 μl of resuspensions of these biofilms diluted in 10 ml of medium were subsequently incubated in blood agar to determine the presence of viable bacteria. The results showed that over 85 CFUs were detected on the blood agar plates of the AgNO_3_, F127-AgNPs-1, and AgNPs-2 subgroups, but few colonies were detected in other subgroups (data not shown). In summary, the order of the complete bactericidal values of these AgNPs subgroups is: F127-AgNPs-3 (3.425 μg/ml) < PVP-AgNPs-2 (7.86 μg/ml) < P2000-AgNPs-3 (14.865 μg/ml) < PVP-AgNPs-3 (17.5 μg/ml) < P123-AgNPs-3 (21.27 μg/ml) < P400-AgNPs-3 (21.85 μg/ml) < P-SH5000-AgNPs-3 (25.6 μg/ml) < P103-AgNPs-3 (31.14 μg/ml) < SDF (45 μg/ml), and undetermined AgNO3 (> 9 μg/ml) < F127-AgNPs-2 (> 41.5 μg/ml) < AgNPs-3 (> 138.9 μg/ml).

### Cytotoxicity of AgNPs and P-AgNPs

**FIGURE 7:**
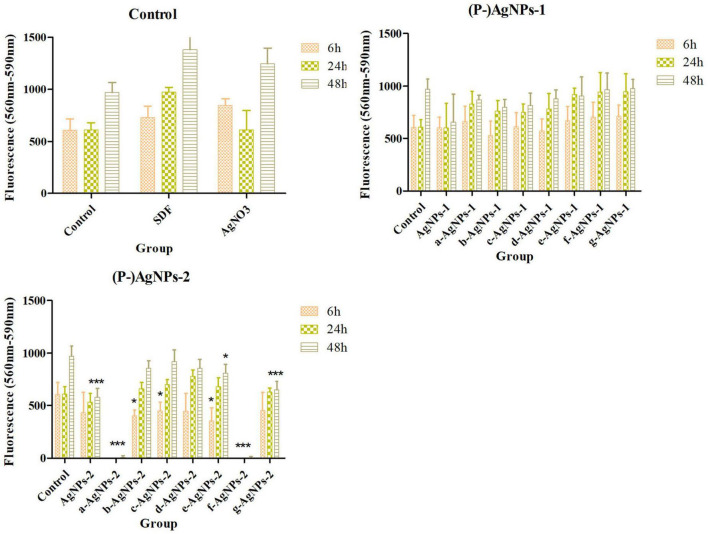
Cytotoxicity of AgNPs-1/2 and P-AgNPs-1/2 on human gingival fibroblasts at 6, 24, and 48 h. “*” *P* < 0.05, “**” 0.05 < *P* < 0.005, “***” *P* < 0.001.

Cytotoxicity assay results of AgNPs and P-AgNPs on HGF are displayed in Figure 7. At MIC level, no significant cytotoxic effects were detected in AgNPs-1 and P-AgNPs-1 subgroups compared with control groups. However, in AgNPs-2 and P-AgNPs-2 groups, P400- and P123- subgroups displayed significant cytotoxicity in 6 h (*P* < 0.001), and cytotoxicity of P400- subgroups appeared as early as 30 min (data not shown). P2000-, P-SH5000- and P103- subgroups also displayed significant cytotoxic effect at 6 h (*P* = 0.006, 0.035, 0.009, respectively), which were significantly weaker than those of the P400- and P123- subgroups, but not at 24 and 48 h. AgNPs-2, F123-AgNPs-2, and F127-AgNPs-2 displayed significant (*P* < 0.001, *P* = 0.023, *P* < 0.001) cytotoxic effects at 48 h.

## Discussion

In the present study, AgNPs and P-AgNPs with spherical morphology, size ranging from 10 to 40 nm, and opposite surface charges were successfully synthesized and confirmed by UV-Vis absorption, DLS and TEM. Results of the comparative analysis show the physical properties of AgNPs-1/2 are not significantly altered by polymer functionalization, but their surface chemical properties have been modified, which brings a significant impact on the biological properties of AgNPs-1/2. However, not all of the polymer-functionalization can improve their biological properties.

Regarding the assessment of physical properties, the complementary combined use of UV-Vis, DLS, and TEM in the present study confirmed the nano dimension and spherical structure of AgNPs and P-AgNPs (Mourdikoudis et al., 2018). The peak values of all AgNPs and P-AgNPs were between 390 and 410 nm, which is the characteristic plasmon resonance absorption peak of typical spherical silver nanoparticles (Udaya et al., 2010). The hydrated size of P-AgNPs increased significantly by more than 25 nm (50–130 nm). However, the TEM results show the dimension of all P-AgNPs-1/2 nanoparticles is between 10 and 40 nm. This indicates that the physical dimension of P-AgNPs does not increase significantly while the increased hydrated size is associated with the coating of polymer. The comparative analysis of the hydrated size of AgNPs-1/2 and P-AgNPs-1/2 shows that the increase in hydrated particle size is not consistent with the molecular weight of polymer. This may be related to the efficiency of polymer adsorption on the surface of AgNPs (Chauhan et al., 2019). Based on the results of the present study, P-SH5000 and PVP have the strongest adsorption capability to AgNPs. Nevertheless, polymer functionalization does not significantly alter the physical dimension of nanoparticles.

In the present study, polymer functionalization did not change the surface charge properties of AgNPs-1/2 but affected their stability. In general, the ζ-potential of a solution is an indicator of the stability of the colloidal dispersion and a solution with an absolute value over 20 is considered stable and dispersed (Mourdikoudis et al., 2018). Hence, the AgNPs-1/2 in this study were stable solutions but the incorporation of polymer seems to negatively affect the stability of AgNPs, especially PEG400-AgNPs-1. To assess the stability of AgNPs, we conducted continuous observation and UV-Vis test in the 28th week. Only AgNPs-2 showed visible agglomeration and precipitation at the 16th week, even though its original ζ-potential was + 46. All other AgNPs and (P-)AgNPs were still spherical nanoparticles at the 28th week (390–410 nm. data not shown). This indicates that polymer functionalization improves the stability of the positively charged AgNPs but does not affect the stability of the negatively charged AgNPs within 28 weeks. However, since ζ-potential value is related to the concentration of solution (Baldassarre et al., 2015; Mourdikoudis et al., 2018), the detected value only reflects the stability of the diluted AgNPs solution to a certain extent. Therefore, we suggest to combine the ζ-potential test and other tests, such as UV-Vis, in longitudinal observations to evaluate the stability of AgNPs.

The Raman spectroscopy studies of (P-)AgNPs qualitatively demonstrate the attachment of polymers on the surface of AgNPs, which brings about modifications of their surface chemical properties. The band of AgNPs-2 and P-AgNPs-2 at 234 cm^–1^ is generally attributed to the stretching vibration of Ag-O bond (Martina et al., 2012). All the AgNPs samples showed board and intense bands at ca. 1,355 and 1,580 cm^–1^. These bands are similar to D/G bands of graphene oxide (Hamzah et al., 2017), which mainly attributed the absorption of carbon polymerized segments on silver (Oliani et al., 2017). The polymer bands ranging from 1,000 to 1,500 cm^–1^ and ca. 2,900 cm^–1^ represent their C-C stretch and CH2 twist (Hsu et al., 2021). The broad and sharp band of P-AgNPs at ca. 500, 650, and 2,900 cm^–1^ may be caused by the presence of polymer vibration. In addition, the bands between 200 and 400 cm^–1^ indicate the emergence of metal-thiol vibrational bond (Rodríguez-Zamora et al., 2022), which identify the attachment of PEG-SH 5000 on AgNPs.

Many researches have shown that the nanometer size (Raza et al., 2016), surface charge (Gottenbos et al., 2001; Abbaszadegan et al., 2015b) and surface chemical (dos Santos et al., 2012; Tiwari et al., 2017; Barrios-Gumiel et al., 2019; Yin et al., 2020) of AgNPs are related to their antibacterial property. In the present study, a negative correlation between size and antibacterial capability was only found in AgNPs-1 and AgNPs-2 without polymer functionalization. This indicates that after polymer functionalization, the modified surface chemical property, rather than particle dimension, is the priority effect factor on the antibacterial property (dos Santos et al., 2012; Tiwari et al., 2017; Barrios-Gumiel et al., 2019; Yin et al., 2020). In addition, the positively charged AgNPs, especially the polymer-functionalized AgNPs in the present study, generally exhibited stronger antibacterial activity than the negatively charged AgNPs over a short period of time. This is highly correlated with the electrostatic interactions between the positively charged materials and the negatively charged surfaces of bacteria (Wilhelm et al., 2020). However, in the present study the negatively charged AgNPs after polymer functionalization, such as with PVP, also exhibited similar or even better antibacterial activity. We speculate that this results from the modification of surface chemistry. PVP has good affinity on cell membrane and cytoplasm (Rackur, 1985), and it increases the connection between membrane and AgNPs through other chemical bonds or synergy effects (Jena et al., 2021), thereby increasing the chance of silver attaching to cells. The specific mechanism remains to be explored in future studies.

Since bacterial biofilm plays a more important role in human health and disease than suspended bacteria (Jamal et al., 2018; Vestby et al., 2020), it is more important to evaluate the antibiofilm effect of AgNPs. It has been found that the initial partition of AgNPs on biofilm interface is controlled by electrostatic interaction (Desmau et al., 2018). This explains why all the positively charged AgNPs and P-AgNPs in the present study displayed good bactericidal effect against *P. gingivalis* biofilm. This kind of electrostatic interaction enhances the attachment of AgNPs and further causes alteration of cell permeability, leading to leakage of intracellular components and cell death (Rabea et al., 2003; Desmau et al., 2018). In addition, the negatively charged AgNPs in the present study, PVP-AgNPs-1 and F127-AgNPs-1, also showed a significant antibiofilm effect against *P. gingivalis* biofilm. We speculate that the enhanced attachment of AgNPs to cells exerts a crucial role in the antibiofilm process as both PVP and F127 have good affinity for cells (Rackur, 1985; Khattak et al., 2005). That is to say, enhancing the attachment of AgNPs on cell membrane by electrostatic interaction or surface chemical modification is an effective method to improve the antibiofilm activity of AgNPs (Kumariya et al., 2015).

Cytotoxicity is another aspect that needs to be evaluated before clinical application of AgNPs. Cytotoxicity of AgNPs is associated with many factors, including nanoparticle dimension, shape, surface charge and surface chemical (El Badawy et al., 2011; Gliga et al., 2014; Silva et al., 2014; Pang et al., 2016). In the present study, surface charge and surface chemical were the only two variables studied, both of which are risk factors affecting the cytotoxicity of AgNPs. The positively charged AgNPs in the present study have higher cytotoxicity and after polymer functionalization, most of the positively charged AgNPs displayed more obvious cytotoxicity. According to the known action mechanism of AgNPs (Pareek et al., 2018; Liao et al., 2019), both the initial attachment of AgNPs to cell membrane and the following release of Ag^+^ ions influence their cytotoxicity, including the subsequent intracellular penetration, reactive oxygen species (ROS), free radical generation, DNA damage and protein denaturation (El Badawy et al., 2011; Nguyen et al., 2016). Both electrostatic interaction and surface chemical modification can enhance the attachment of AgNPs on cell membrane and cellular uptake, resulting in stronger cytotoxicity (Treuel et al., 2014; Malysheva et al., 2021). However, there is also polymer functionalization in the present study, that is PVP, which did not increase the cytotoxicity of positively charged AgNPs. We speculate that this is related to the low treatment concentration of PVP-AgNPs-2. Based on the “Trojan-horse” mechanism, it is postulated that the cytotoxicity of AgNPs is associated with the release of toxic ions in intracellular environments, and both intracellular ROS and acidic condition of lysosomal cellular compartment can induce the reaction with AgNPs to form more Ag^+^ ions (Sabella et al., 2014; Hsiao et al., 2015). This mechanism supports that the cytotoxicity of AgNPs is dose-dependent, involving the release of Ag^+^ ions (Pareek et al., 2018; Liao et al., 2019). Low doses of silver reduce cytotoxicity by inducing protective autophagy in cells (Li et al., 2020). However, the F127-AgNPs-2 in the present study, which is the lowest treatment concentration subgroup, showed cytotoxicity at 48 h. We attribute this to the surface chemical modification caused by the F127 polymer which affects the attachment, release and accumulation of silver on the cell surface, thereby causing long-term cytotoxicity. In summary, the lower the working concentration of AgNPs, the higher the potential for low cytotoxicity is, and surface chemical also is a significant effect factor.

It should be noted that none of the AgNPs in the present study showed no cytotoxicity at concentrations capable of exerting antibiofilm activity. All the positively and negatively charged AgNPs and P-AgNPs showed significant cytotoxicity on HGF at concentrations higher than 2.5* MIC. We speculate that there is a certain positive correlation between the antibacterial capability of AgNPs and its cytotoxicity. Khalil et al. (2019) also found that the antibacterial material with lower MIC value in their study exhibited higher cytotoxicity. Hence, we should find a balance between the antibacterial property and the cytotoxicity of antibacterial materials for application. Besides, we should not ignore the synthesis method of AgNPs, because some studies report that green synthesized AgNPs show better biocompatibility (Selvakumar et al., 2018; Alavi et al., 2022). In summary, for clinical application, further investigation on how to reduce the cytotoxicity of (P-)AgNPs at the antibiofilm concentration level is needed.

## Conclusion

Polymer functionalization, as carried out in the present study, does not significantly alter the physical properties of AgNPs, including their size and surface charge. The alteration in biological properties mainly attributes to the modification in surface chemical of P-AgNPs. In the present, some polymer-functionalization, especially PVP, significantly enhanced the antibacterial and antibiofilm effect of AgNPs, which varied with the surface charge. However, the cytotoxicity of AgNPs was not significantly improved by polymer-functionalization, especially at the antibiofilm concentration level.

## Data availability statement

The datasets presented in this study can be found in online repositories. The names of the repository/repositories and accession number(s) can be found in the article/Supplementary material.

## Author contributions

MZ conceived the project, performed the entire experiment, analyzed and interpreted the data, and was a major contributor in writing the manuscript. EL conceived the project, analyzed and interpreted the data, and revised the manuscript. Both authors read and approved the final manuscript.
