# Supplementary material for: Compare the physicochemical and biological properties of engineered polymer-functionalized silver nanoparticles against Porphyromonas gingivalis
Source: Front Microbiol. 2022 Sep 8;13:985708. doi: 10.3389/fmicb.2022.985708 (PMC9493255; doi:10.3389/fmicb.2022.985708)

Compare the physicochemical and biological properties of engineered polymer-functionalized silver nanoparticles against *Porphyromonas gingivalis*

**Meng Zhang ^1^, Edward CM. Lo ^1*^**

1 Faculty of Dentistry, The University of Hong Kong, 34 Hospital Road, Sai Ying Pun, Hong Kong, 999077, China.

*** Correspondence:**

Edward Chin Man Lo, hrdplcm@hku.hk

**Keywords:** Antibacterial, Antibiofilm, Cytotoxicity, Polymers, *Porphyromonas gingivalis*, Silver nanoparticles

Table S1. The detail ratios and doses of AgNPs-1/2 and polymers for the synthesis of the Polymer-functionalized AgNPs.

| AgNPs-1 | Ag^+^ molar | 4.17 μmol |  | AgNPs-2 | Ag^+^ molar | 6.35 μmol |  |
| --- | --- | --- | --- | --- | --- | --- | --- |
|  | Ratio  (Ag^+^:Polymer) | Weight  (mg) | Volume  (ml) |  | Ratio (Ag^+^:Polymer) | Weight  (mg) | Volume (ml) |
| PEG 400 | 1:4 | 6.7 | 10 | PEG 400 | 1:4 | 10 | 10 |
| PEG 2000 | 1:10 | 83.4 | 10 | PEG 2000 | 1:10 | 127 | 10 |
| PEG-SH 5000 | 1:1 | 20 | 10 | PEG-SH 5000 | 1:1 | 30 | 10 |
| PVP 10000 | 1:10 | 417 | 10 | PVP 10000 | 1:10 | 635 | 10 |
| P 103 | CMC 0.1 mg/ml | 5 |  | P 103 | CMC 0.1 mg/ml | 2 |  |
| P 123 | CMC 0.052 mg/ml | 2.6 |  | P 123 | CMC 0.052 mg/ml | 1 |  |
| F 127 | CMC 1 mg/ml | 50 |  | F 127 | CMC 1 mg/ml | 20 |  |

Figure S1. Raman spectrum of (A) silicon disc, AgNO3, polymers, and (B) P103-AgNPs-2.


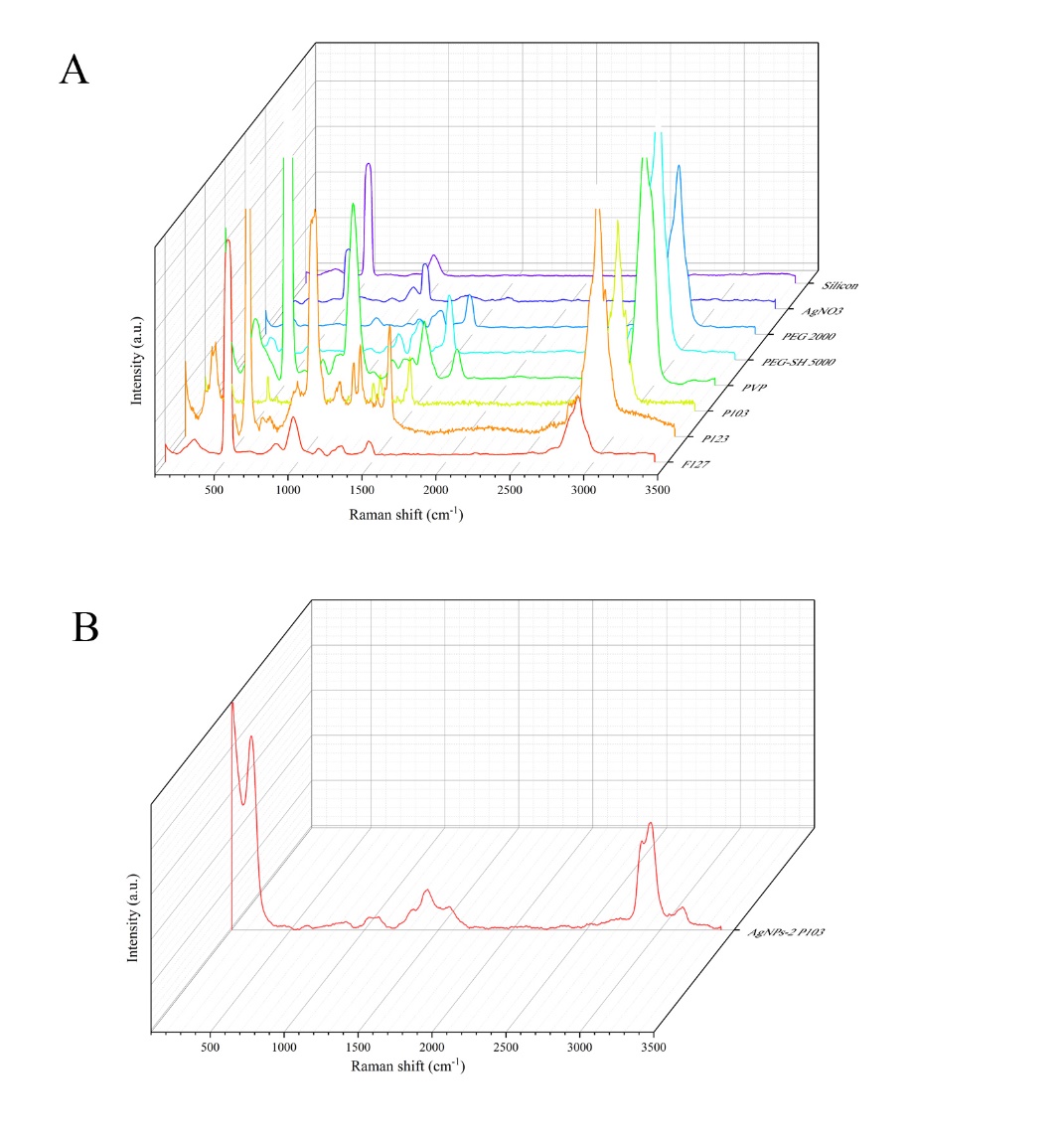

Supplement: Supplementary file 1 [file Table_1.DOCX]
